# Lateral Facilitation between Primary Mechanosensory Neurons Controls Nose Touch Perception in *C*. *elegans*

**DOI:** 10.1016/j.neuron.2011.02.046

**Published:** 2011-04-28

**Authors:** Marios Chatzigeorgiou, William R. Schafer

**Affiliations:** 1 Cell Biology Division, MRC Laboratory of Molecular Biology, Hills Road, Cambridge CB2 2QH, UK

## Abstract

The nematode *C*. *elegans* senses head and nose touch using multiple classes of mechanoreceptor neurons that are electrically coupled through a network of gap junctions. Using in vivo neuroimaging, we have found that multidendritic nociceptors in the head respond to harsh touch throughout their receptive field but respond to gentle touch only at the tip of the nose. Whereas the harsh touch response depends solely on cell-autonomous mechanosensory channels, gentle nose touch responses require facilitation by additional nose touch mechanoreceptors, which couple electrically to the nociceptors in a hub-and-spoke gap junction network. Conversely, nociceptor activity indirectly facilitates activation of the nose touch neurons, demonstrating that information flow across the network is bidirectional. Thus, a simple gap-junction circuit acts as a coincidence detector that allows primary sensory neurons to integrate information from neighboring mechanoreceptors and generate somatosensory perception.

## Highlights

► *C. elegans* polymodal nociceptors sense heat, harsh head touch and gentle nose touch ► Harsh head touch is sensed by DEG/ENaC channels ► Nose touch is facilitated by electrical synapses with nose mechanoreceptor neurons ► Information flow in nose touch network is bidirectional

## Introduction

Somatosensory circuits, which gather sensory information from the skin and body surface, are a feature of most animal nervous systems. A patch of skin typically contains multiple classes of primary somatosensory neurons with dendrites responding to distinct sensory modalities. Somatosensory circuits include thermosensory neurons responding to temperature, touch neurons responding to gentle pressure or motion, proprioceptors responding to body posture, and nociceptors responding to harsh, body-damaging stimuli. Touch neurons, proprioceptors, and nociceptors share the property that their activities are controlled by mechanical force.

Most, if not all, primary mechanosensory neurons sense force using ion channels that are directly mechanically gated. Many of these channels, particularly in invertebrates, appear to come primarily from one of two protein superfamilies: the TRP channels, and the DEG/ENaC channels (Garcia-Anoveros and Corey, 1997; Goodman et al., 2004). TRP channels are nonspecific cation channels composed of subunits with six transmembrane α helices. At least some TRP channels appear to be sufficient by themselves to produce touch- or stretch-evoked currents (Christensen and Corey, 2007). In addition, TRP channels can be activated by G protein signaling, which has been implicated in other sensory transduction processes including taste, vision, and olfaction (Kahn-Kirby and Bargmann, 2006). In contrast, DEG/ENaC channel subunits have two transmembrane α helices and form channels that are permeable to sodium and, in some cases, calcium (Bounoutas and Chalfie, 2007). Both families have been implicated in mechanosensory transduction in invertebrates as well as vertebrates.

The process of mechanosensation has been extensively studied in genetically tractable organisms such as *C*. *elegans* (Arnadóttir and Chalfie, 2010). Touch is an important sensory modality for *C*. *elegans*; indeed, over 10% of the neurons in the adult hermaphrodite are thought to be mechanoreceptors responding to external touch stimuli (White et al., 1986). The best studied of these are the five neurons (ALML, ALMR, AVM, PLML, and PLMR) that sense gentle body touch. These cells sense low-threshold mechanical stimuli using a mechanotransduction complex whose core components include the DEG/ENaC channel proteins MEC-4 and MEC-10 and the stomatin MEC-2 (Driscoll and Chalfie, 1991; O'Hagan et al., 2005). Activation of the ALM and AVM anterior touch neurons triggers a change from forward to backward movement; this escape response appears to depend primarily on gap junctions between the mechanoreceptor neurons and the backward-command interneurons that potentiate backward locomotion (Chalfie et al., 1985). Conversely, activation of PLM posterior body touch receptors activates forward-command interneurons that promote accelerated forward locomotion. An additional pair of neurons in the body, the PVD multidendritic nociceptors, are required to generate escape responses to harsh body touch (Way and Chalfie, 1989).

*C*. *elegans* also respond to touch stimulation on the nose. When an animal collides with an object head-on, it reverses direction in a manner similar to the anterior touch escape reflex. As many as 20 neurons with sensory endings in or around the nose have been implicated by morphological or functional criteria as potential nose touch mechanoreceptors. Cell ablation experiments indicated that loss of either of two neuron pairs, the ASH and FLP neurons, causes a partial reduction in nose touch response, and elimination of both classes results in a strong nose touch defect (Kaplan and Horvitz, 1993). These results led to the conclusion that ASH and FLP are the primary sensory neurons involved in the nose touch escape reflex. The ASH neurons are polymodal nociceptors that respond to chemical and osmotic stimuli in addition to nose touch (Kaplan and Horvitz, 1993), and their responses to all these stimuli are dependent on the TRPV channel OSM-9 (Colbert et al., 1997). The FLPs have highly branched multidendritic arbors that surround the animal's head, suggesting that they may also be nociceptors (Hall and Altun, 2008; Albeg et al., 2011). The FLPs express the DEG/ENaC channel MEC-10 (Huang and Chalfie, 1994; Chatzigeorgiou et al., 2010b) as well as the OSM-9 TRPV channel (Colbert et al., 1997), though, to our knowledge, the effects of these molecules on mechanosensation in the FLPs have not been reported.

**Figure 1:**
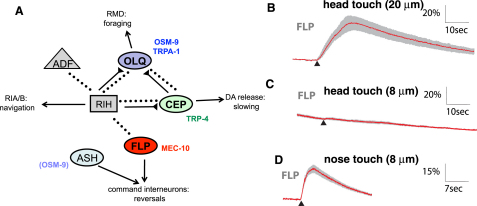
The FLP Neurons Respond to Harsh Head Touch and Gentle Nose Touch (A) Connections between nose touch mechanoreceptors. Shown are synaptic and electrical connections involving FLP and other nose touch mechanoreceptors. Gap junctions are indicated by dashed lines, chemical synapses by solid lines, with triangles signifying presynaptic terminals. Known or hypothesized outputs of sensory neurons (Alkema et al., 2005; Hart et al., 1995; Sawin et al., 2000) are indicated. Also indicated are sites of transgenic rescue for genes involved in nose touch behavior and nose-touch-evoked calcium transients in FLP as determined in this study. MEC-10 acts cell autonomously in the FLPs; OSM-9 acts in the OLQs, and TRP-4 acts in the CEPs and other dopaminergic mechanoreceptors. OSM-9 also acts in the ASH neurons to promote nose touch behavior, though expression here does not affect neural responses to nose touch in FLP (see Figure 3). (B–D) Averaged calcium responses to harsh head touch (B), gentle head touch (C), and gentle nose touch (D). Each red trace represents the average percentage change in R/R_0_ for the indicated genotype, where R is the fluorescence emission ratio at a given time point, and R_0_ is its initial value. The number of individual recordings averaged for each stimulus condition was 24 (harsh head touch), 21 (gentle head touch), and 12 (gentle nose touch). Gray shading indicates SEM of the mean response. Scale bars are indicated.

Additional neurons have been implicated as nose touch mechanosensors, though their importance in nose touch avoidance behavior is less well established (Figure 1A). The four OLQ neurons have ciliated endings in the outer labial sensilla that suggest a function as mechanoreceptors. Ablations of the OLQs alone have little effect on nose touch escape responses, though they enhance the defects of ASH and FLP ablations (Kaplan and Horvitz, 1993). However, the OLQs have been implicated in another nose touch-related behavior, the suppression of lateral “foraging” movements of the head by nose or anterior body touch (Driscoll and Kaplan, 1997; Hart et al., 1995; Alkema et al., 2005; Kindt et al., 2007b). OLQ ablations also affect the rate and amplitude of foraging in unstimulated animals, suggesting a role in mechanosensory feedback for this behavior. Nose touch evokes calcium transients in the OLQs, which are affected by mutations in the TRPA channel *trpa-1* (Kindt et al., 2007b). The four CEP neurons also have sensory cilia in the nose that indicate a role as mechanoreceptors. Although ablations of the CEPs affect neither nose touch avoidance nor foraging behaviors, they do act with the other dopaminergic neurons to mediate a slowing response to a bacterial lawn, which appears to involve mechanical detection of bacteria (Sawin et al., 2000). Gentle nose touch evokes neural responses in CEP that require the cell-autonomous activity of the TRPN channel TRP-4 (Kindt et al., 2007a; Kang et al., 2010). Thus, both the OLQ and CEP neurons appear to sense nose touch; however, their absence primarily affects foraging and slowing behaviors rather than nose touch avoidance.

In this study, we investigate the circuit for *C*. *elegans* nose touch avoidance in more detail using a combination of neuroimaging and behavioral analysis. We find that the FLP neurons are polymodal nociceptors that respond to harsh touch as well as heat. In addition, the FLPs respond to gentle touch applied to the more restricted region of the nose. Whereas harsh head touch is dependent only on the cell-autonomous activity of a MEC-10-containing DEG/ENaC complex, gentle nose touch also requires *mec-10*-independent contributions from other nose touch neurons that are coupled to FLP through gap junctions. Activation of the gentle nose touch neurons thus acts in a circuit-dependent manner to facilitate low-threshold responses in the otherwise high-threshold nociceptor neurons.

## Results

### The FLP Multidendritic Nociceptors Respond to Harsh Head Touch, Gentle Nose Touch, and Heat

The FLP neurons have been implicated by ablation studies in nose touch sensation. In addition, they have a multidendritic morphology characteristic of polymodal nociceptors, suggesting that they might respond to touch stimuli on other parts of the head or to other noxious stimuli such as extreme temperatures. To assess the sensory responses of the FLP neurons, we used a transgenic line, *ljEx19*, that expressed the calcium-sensitive fluorescent protein YC2.3 in the FLP neurons under the control of the *egl-46* promoter (Wu et al., 2001; Chatzigeorgiou et al., 2010b). Nose touch behavior was normal in this line (Figure S1A available online); thus, we applied harsh and gentle touch stimuli by pressing a rounded glass probe to the side of the head in the region of the FLP dendritic lattice (Figure S1B) and imaged calcium transients evoked in the FLP cell body. For mechanical stimuli applied directly to the nose, we observed that a small (8 μM) displacement motion stimulus evoked a robust calcium transient similar in dynamics to responses seen in other *C*. *elegans* nose touch neurons (Kindt et al., 2007a, 2007b). In contrast, small displacement stimuli applied to the side of the head did not evoke calcium transients in FLP. However, large and long-lasting calcium transients could be evoked by a mechanical stimulus of large (20 μM) displacement and high velocity (Figures 1B–1D). Thus, the FLP neurons exhibited distinct responses to gentle nose touch and harsh head touch. Since the other multidendritic neurons in *C*. *elegans*, the PVDs, exhibit a TRPA-1-dependent response to cold shock (Chatzigeorgiou et al., 2010b), we also tested FLP responses to temperature changes. We observed that rapid increases in temperature from 20°C to 35°C led to robust calcium transients, indicating that the FLP neurons respond to noxious heat (Figure S2A). Thus, the FLP neurons appear to be polymodal nociceptors responding to heat, harsh head touch, and gentle nose touch.

**Figure 2:**
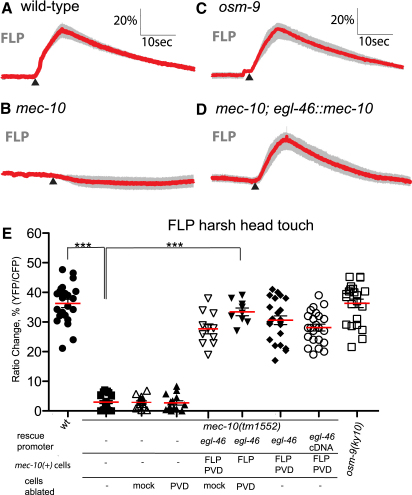
MEC-10 Is Required Cell Autonomously for FLP Harsh Touch Response Averaged responses of wild-type (A), *mec-10(tm1552)* (B), *osm-9(ky10)* (C), and *mec-10(tm1552); egl-46::mec-10* (D) to harsh head touch stimulation in FLP. Each red trace represents the average percent change in R/R_0_ for 21 (wild-type, *osm-9*, and *mec-10; ljEx220[egl-46::mec-10genomic]*) or 14 (*mec-10*) individual recordings. Gray shading indicates the SEM. None of these genotypes visibly altered the morphology of FLP, or the expression pattern of the cameleon transgene. (E) Scatter plot of peak calcium responses for each genotype. Statistical significance (^∗∗∗^p < 0.0005) is according to the Mann-Whitney U rank sum test. Also shown are data for *mec-10(tm1552); egl-46::mec-10cDNA* (n = 23), PVD-ablated *mec-10* (n = 14) and *mec-10(tm1552); egl-46::mec-10* (n = 9), and mock-ablated *mec-10* (n = 11) and *mec-10(tm1552); egl-46::mec-10* (n = 14). These results, together with those in Figure S3, demonstrate that the transgenic rescue results specifically from expression of *mec-10(+)* in FLP, and not PVD.

**Figure 3:**
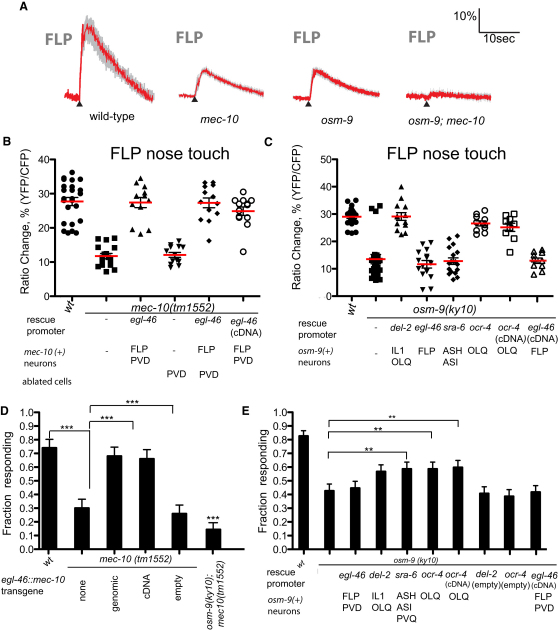
MEC-10 Is Required Cell Autonomously and OSM-9 Nonautonomously for FLP Nose Touch Response (A) Averaged responses of wild-type, *mec-10(tm1552)*, *osm-9(ky10)*, and *osm-9(ky10); mec-10(tm1552)* to gentle nose touch stimulation in FLP. Each red trace represents the average percentage change in R/R_0_ for wild-type (n = 24), *mec-10* (n = 22), *osm-9* (n = 22), and *osm-9;mec-10* (n = 13) individual recordings; gray shading indicates SEM. (B and C) Scatter plot of peak calcium responses for each genotype. In addition to the genotypes in (A), we analyzed *mec-10(tm1552); egl-46::mec-10(genomic)* (n = 13) and *mec-10(tm1552); egl-46::mec-10(cDNA)* (n = 13) rescue lines in (B), and *osm-9(ky10)* rescue lines expressing *osm-9(+)* under the *del-2* (genomic fragment, n = 13), *egl-46* (genomic fragment, n = 15; cDNA n = 10), *sra-6* (genomic fragment, n = 17), or *ocr-4* (genomic fragment, n = 10; cDNA, n = 9) promoters in (C). For (B), also shown are data for *mec-10* mutant (n = 13) and rescue animals (n = 13) in which the PVD harsh body touch neurons have been eliminated by laser ablation; these results demonstrate that the transgenic rescue results specifically from expression of *mec-10(+)* in FLP. (D and E) Effects of *mec-10* and *osm-9* on nose touch escape behavior. For all genotypes at least 50 animals were scored for reversals following nose touch stimulation. Statistical significance (^∗∗^p < 0.001, ^∗∗∗^p < 0.0001) is according to the Student's t test.

The FLP neurons express the DEG/ENaC channel MEC-10, which contributes to mechanotransduction channels in other *C*. *elegans* neurons (Huang and Chalfie, 1994; O'Hagan et al., 2005; Chatzigeorgiou et al., 2010a; Chatzigeorgiou et al., 2010b). We therefore tested the effect of a *mec-10* loss-of-function mutation on FLP responses to sensory stimuli. For harsh head touch, the *mec-10(tm1552)* mutant was strongly defective in touch-evoked calcium transients (Figures 2A and 2B). This defect was rescued by expressing the wild-type *mec-10(+)* allele under the control of the *egl-46* promoter (which is FLP specific when the PVD body nociceptors are eliminated; see Figure S3), indicating that MEC-10 functions cell autonomously in the FLP neurons (Figures 2D and 2E). In contrast, *mec-10(tm1552)* did not affect FLP responses to heat (Figure S2). Thus, MEC-10 does not generally disrupt FLP physiology or excitability, and appears to function specifically in the process of mechanosensation. Finally, we observed that *mec-10(tm1552)* animals showed a partial though significant reduction in the magnitude of the calcium transient evoked by gentle nose touch (Figures 3A and 3B). This defect was rescued by an *egl-46::mec-10(+)* transgene, indicating that the requirement for MEC-10 in FLP nose touch response is cell autonomous (Figure 3B). *mec-10(tm1552)* animals also showed a behavioral defect in nose touch escape response, which was rescued by *egl-46::mec-10(+)* (Figure 3D). Thus, whereas responses to harsh head touch are completely MEC-10 dependent, gentle nose touch responses are only partially dependent on MEC-10.

### OSM-9 TRP Channels Function Nonautonomously in FLP Mechanosensation

To identify the molecules contributing to the MEC-10-independent component of the nose touch response, we assayed additional candidate sensory transduction mutants. In addition to MEC-10, another potential mechanotransduction channel is expressed in the FLP neurons: the TRPV channel OSM-9 (Colbert et al., 1997). To determine whether OSM-9 could contribute to the nose touch response remaining in *mec-10(tm1552)* mutant animals, we imaged FLP responses to nose touch in *osm-9(ky10)* single mutant and *osm-9(ky10); mec-10(tm1552)* double mutant animals. We observed that a null mutation in *osm-9* led to a significant reduction in nose-touch-evoked calcium transients in FLP (Figure 3A), though it had no effect on response to harsh head touch (Figure 2C) and did not alter (Tobin et al., 2002) FLP morphology or reporter expression (Figure S4). Furthermore, an *osm-9(ky10); mec-10(tm1552)* double mutant showed virtually no significant calcium increase in response to nose mechanosensory stimulation in FLP (Figure 3A). These results indicate that MEC-10 and OSM-9 contribute additively to the mechanosensory response to nose touch in FLP.

We next carried out cell-specific rescue experiments to determine whether OSM-9, like MEC-10, functions cell autonomously in the FLP neurons. Unexpectedly, expression of *osm-9(+)* under the FLP-specific *egl-46* promoter did not rescue the nose touch phenotype in FLP (Figures 3C and 3E), though its ability to rescue a heat response defect indicated that it was functionally expressed in the FLP neurons (Figures S2C and S2D). Likewise, expression of *osm-9(+)* in the ASH nociceptor neurons did not restore nose touch responses in the FLP neurons, though it did rescue the ASH-mediated *osm-9* osmotic avoidance defect (Figure S5). However, an *osm-9(+)* cDNA or genomic fragment robustly rescued the FLP nose touch defect (Figures 3C and 3E) when expressed under the control of either the *del-2* promoter fragment, specific for the OLQ and IL1 labial mechanoreceptors (Kindt et al., 2007b), or the OLQ-specific *ocr-4* promoter (Tobin et al., 2002). These results suggest that OSM-9 functions in the OLQ labial mechanoreceptors to indirectly promote FLP nose touch responses.

**Figure 4:**
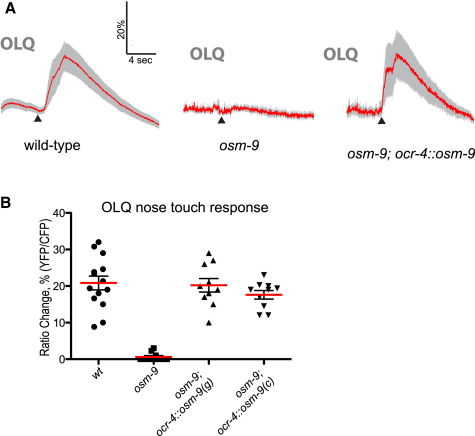
Effect of *osm-9* on OLQ Nose Touch Responses (A) OSM-9 is required cell autonomously for OLQ nose touch response. Shown are averaged responses of wild-type (n = 14), *osm-9(ky10)* (n = 10), and *osm-9(ky10); ocr-4::osm-9(genomic)* (n = 10) to nose touch stimulation in OLQ. Gray shading indicates the SEM. None of these genotypes visibly altered the morphology of OLQ, or the expression pattern of the cameleon transgene (see Figure S4). (B) Scatter plot of peak OLQ calcium responses for *osm-9* genotypes. In addition to the strains shown in (A), we imaged ten animals in which *osm-9(ky10)* was rescued by an *ocr-4::osm-9* cDNA transgene.

The OLQ neurons have been shown previously to respond to nose touch. To determine whether OSM-9 is required cell autonomously in OLQ for nose touch responses, we imaged nose-touch-evoked calcium transients in OLQ using a previously described *ocr-4::YCD3* cameleon line (Kindt et al., 2007b). We found that calcium transients were robustly evoked by gentle nose touch responses in the wild-type OLQ neurons but were completely absent in the *osm-9(ky10)* mutant background (Figures 4A and 4B). This defect could be rescued by cell-specific expression of *osm-9(+)* under the OLQ-specific *ocr-4* promoter (Figures 4A and 4B). Thus, OSM-9 is required cell autonomously for the OLQs to respond to nose touch. This result suggested the possibility that gentle nose touch sensation by OLQ might indirectly promote nose touch responses in FLP.

### A Network Centered on the RIH Interneuron Facilitates FLP Nose Touch Responses

**Figure 5:**
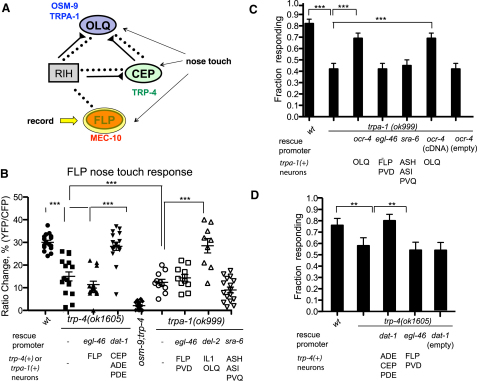
Effects of OLQ and CEP Sensory Inputs on Nose Touch (A and B) Effects of *trp-4* and *trpa-1* on FLP nose touch responses. As diagrammed in (A), we imaged FLP nose touch responses in animals carrying mutations affecting the OLQ (*trpa-1*) or CEP (*trp-4*) neurons. Shown in (B) is a scatter plot of peak calcium responses (percentage change in R/R_0_) for 16 wild-type, 16 *trp-4(ok1605)*, 16 *trp-4; egl-46::trp-4*, 16 *trp-4; dat-1::trp-4*, 16 *osm-9(ky10); trp-4(ok1605)*, 11 *trpa-1(ok999)*, 11 *trpa-1; egl-46::trpa-1*, 9 *trpa-1; del-2::trpa-1*, and 16 *trpa-1; sra-6::trpa-1* animals. Statistical significance (^∗∗∗^p < 0.0005) is according to the Mann-Whitney U rank sum test. (C and D) Effect of the *trp-4* and *trpa-1* on nose touch behavior. For all genotypes at least 50 animals were scored for reversals following nose touch stimulation. Statistical significance (^∗∗^p < 0.001, ^∗∗∗^p < 0.0001) is according to the Student's t test.

How might the OLQ mechanoreceptors facilitate nose touch responses in FLP? The FLP and OLQ mechanoreceptors are both linked by gap junctions to RIH (White et al., 1986), an interneuron that also makes gap junctions with the dopaminergic CEP mechanoreceptors and the ADF taste chemoreceptors (Figure 1A). A similar hub-and-spoke network was recently shown to control aggregation behavior in *C*. *elegans* (Macosko et al., 2009). We reasoned that this network might allow the OLQ and CEP neurons to facilitate FLP activity through electrical signaling. Consistent with this hypothesis, we observed that loss-of-function mutations in *trpa-1* (which partially reduce OLQ mechanosensation; Figure S6; Kindt et al. 2007b) led to a reduction in nose-touch-evoked calcium transients in FLP (Figures 5A and 5B). As was the case for *osm-9*, this defect in FLP calcium response as well as the *trpa-1* nose touch avoidance defect was rescued cell extrinsically by expression of the wild-type transgene in OLQ (Figures 5B and 5C). This provides further evidence that the OLQs facilitate FLP nose touch response, possibly through gap junctions with RIH.

The hub-and-spoke hypothesis predicts that the CEP and RIH neurons should also be important for nose touch responses in FLP. We first tested whether the CEP neurons contribute to FLP nose touch responses. Responses to gentle nose touch in the CEP neurons have been shown to require the TRPN channel TRP-4 (Li et al., 2006; Kindt et al., 2007a; Kang et al., 2010). When we imaged nose touch responses in FLP, we observed a significant reduction in the nose-touch-evoked calcium transient in the *trp-4* null mutant (Figure 5B). This defect in FLP calcium response could be rescued by expression of a *trp-4* cDNA in the CEPs under the *dat-1* promoter, but not by expression of *trp-4* in the FLP neurons themselves (Figure 5B). *trp-4* mutants also exhibited a partial defect in nose touch avoidance behavior, which was rescued by functional expression in the CEPs but not the FLPs (Figure 5D). Thus, TRP-4-mediated nose touch responses in CEP, like OSM-9-mediated responses in OLQ, appear to contribute to nose touch responses in FLP. Interestingly, compromising both the OLQ and CEP inputs in an *osm-9; trp-4* double mutant led to a complete loss of nose touch responses in FLP (Figure 5B). These results indicate that the OLQ and CEP neurons function additively to promote responses to small-displacement nose touch stimuli in FLP.

**Figure 6:**
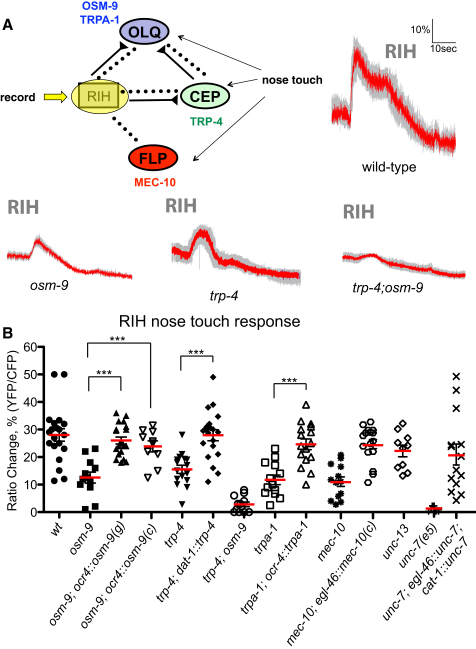
The RIH Interneuron Integrates Responses to Nose Touch (A) Averaged responses of wild-type, *trp-4(ok1605) osm-9(ky10)*, and *osm-9(ky10); trp-4(ok1605)* to nose head touch stimulation in RIH. As diagrammed, we imaged RIH calcium transients in response to nose touch stimulation. Each solid trace represents the average percentage change in R/R_0_ for 20 wild-type, 14 *osm-9(ky10)*, 16 *trp-4(ok1605)*, and 12 *osm-9(ky10); trp-4(ok1605)* individual animals. Gray shading indicates the SEM. None of these genotypes visibly altered the morphology of RIH (data not shown), or the expression pattern of the cameleon transgene (Figure S4). (B) Scatter plot of peak calcium responses for each genotype. In addition to the genotypes in (A), ten *unc-13*, ten *unc-7*, 14 *unc-7; egl-46::unc-7; cat-1::unc-7* ; 13 *trpa-1*, 16 *trpa-1; ocr-4::trpa-1*, 20 *trp-4; dat-1:: trp-4,* 13 *mec-10,* 16 *mec-10; egl-46::mec-10(cDNA),* 20 *osm-9; ocr-4::osm-9(genomic)*, and ten *osm-9; ocr-4::osm-9(cDNA)* individual animals were analyzed. Statistical significance (^∗∗∗^p < 0.0005) is according to the Mann-Whitney U rank sum test.

Our model also predicts that the RIH neurons should be activated by nose touch stimuli in a manner dependent on the OLQ and/or CEP neurons. To test this possibility we used the *cat-1::YCD3* transgenic line, which expresses cameleon in RIH, to measure calcium dynamics following nose touch stimulation. We observed (Figure 6A) that small-displacement nose touch stimuli indeed evoked large calcium transients in RIH. These transients were similar to the sensory neuron transients in magnitude (28% ΔR/R_0_) but were significantly longer in duration, with some responses lasting as long as 25 s. Mutations in *osm-9* or *trpa-1*, which eliminate or reduce OLQ nose touch responses, or in *trp-4*, which eliminate CEP nose touch responses, reduced the nose-touch-evoked transients in RIH and were rescued cell specifically in the appropriate neurons (Figures 6A and 6B). Moreover, a *trp-4; osm-9* double mutant, in which OLQ and CEP nose touch responses were both eliminated, showed virtually no nose-touch-evoked calcium transients in RIH (Figures 6A and 6B). Together, these data indicate that the RIH interneuron is activated by the OLQ and CEP nose touch mechanoreceptor neurons.

**Figure 7:**
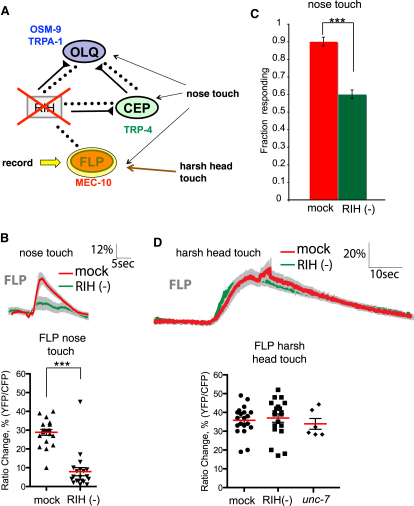
The RIH Network Is Important for FLP Responses to Nose Touch but Not Harsh Head Touch (A) Imaging the effect of RIH ablation on FLP responses to nose touch and harsh head touch. As diagrammed, we recorded calcium transients in FLP following mechanical stimulation in intact and RIH-ablated animals. (B) Responses of wild-type and RIH-ablated animals to nose touch stimulation. Each solid trace represents the average percentage change in R/R_0_ for 24 (mock-ablated, red trace) or 13 (RIH-ablated, green trace) individual recordings. Gray shading indicates SEM of the mean response. Scale bars are indicated above. The green bar indicates the time of the stimulus. Ablation of RIH did not visibly alter the morphology of FLP or RIH, or the expression patterns of the cameleon transgenes. Scatter plot shows peak responses of 20 mock-ablated and 20 RIH-ablated animals. Statistical significance (^∗∗∗^p < 0.0005) is according to the Mann-Whitney U rank sum test. (C) Effect of RIH ablation on nose touch escape behavior. Animals were touched on the nose, and escape responses (reversals) were scored as described. At least 100 animals were tested for each genotype. Statistical significance (^∗∗∗^p < 0.0005) is according to the Student's t test. (D) Responses of wild-type and RIH-ablated animals to harsh head touch stimulation. Each solid trace represents the average percentage change in R/R_0_ for 24 (mock-ablated, red trace) or 13 (RIH-ablated, green trace) individual recordings. Gray shading indicates SEM of the mean response. Scale bars are indicated above. Scatter plot shows peak responses of 20 mock-ablated animals, 20 RIH-ablated animals, and six *unc-7* mutant animals (*unc-7* nose touch responses are in Figure S7).

A third prediction of our model is that the RIH neuron should be required for FLP responses to small-displacement nose touch stimuli. To test this prediction, we eliminated RIH through cell-specific laser ablation, and determined the effect of this lesion on calcium transients in FLP (Figure 7A). We observed that FLP responses to nose touch were greatly reduced in RIH-ablated animals (Figure 7B). Behavioral responses to nose touch were likewise impaired in animals lacking the RIH neuron (Figure 7C). In contrast, FLP responses to harsh head touch were unaffected by RIH ablation (Figure 7D). Thus, the RIH interneuron is specifically important for the activation of the FLP neurons in response to nose touch stimulation. Together, these findings indicate that the RIH interneurons facilitate the flow of sensory information from the OLQ and CEP mechanoreceptors to the FLP nociceptor neurons.

To specifically assess the involvement of electrical signaling, we assayed the responses of mutants defective in the annexin gene *unc-7*, which encodes a major component of gap junctions in many *C*. *elegans* neurons (Starich et al., 1993, 2009). We observed that nose-touch-evoked calcium transients in RIH were nearly completely absent in *unc-7* mutants (Figure 6B; Figure S7). Likewise, nose-touch-evoked calcium transients in FLP were significantly reduced, resembling in magnitude the responses in the RIH-ablated animals (Figure S7); FLP harsh head touch responses, in contrast, were unaffected (Figure S7). *unc-7* loss-of-function mutants showed partial defects in nose touch escape behavior (Figures S7 and S8). These nose touch defects were rescued when a functional *unc-7(+)* transgene was expressed in the nose touch circuit using the *cat-1* (expressed in the CEPs, RIH, and few other neurons) and *egl-46* (expressed in FLP and PVD) promoters (Figure 6B; Figures S7 and S8). *unc-7(+)* expression using either promoter alone did not result in phenotypic rescue (data not shown), suggesting that gap junction formation requires production of the innexin protein in both connected neurons. In contrast, mutations in *unc-13*, which impair synaptic transmission, did not detectably impair RIH nose touch responses (Figure 6B). Together, these results support the hypothesis that signaling in the RIH-centered nose touch circuit is predominantly, if not exclusively, mediated by gap junctions.

### Information Flow through the Nose Touch Network Is Bidirectional

**Figure 8:**
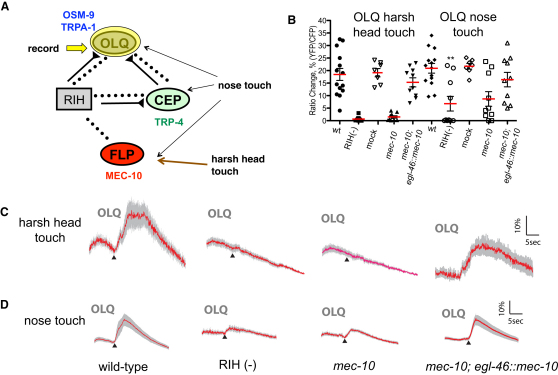
The RIH Network Is Important for OLQ Responses to Nose Touch and Harsh Head Touch (A) Imaging OLQ responses to nose touch and harsh head touch. As diagrammed, we recorded calcium transients in OLQ following mechanical stimulation in wild-type, ablated, and mutant animals. (B) Scatter plot of peak calcium responses to nose touch or harsh head touch in OLQ. For harsh head touch, 14 wild-type, ten RIH-ablated wild-type, seven mock-ablated wild-type, ten *mec-10(tm1552)*, and ten *mec-10(tm1552); egl-46::mec-10(cDNA)* were imaged. For nose touch, 14 wild-type, ten RIH-ablated wild-type, seven mock-ablated wild-type, ten *mec-10(tm1552)*, and ten *mec-10(tm1552); egl-46::mec-10(cDNA)* were imaged. Statistical significance (^∗∗∗^p < 0.0005) is according to the Mann-Whitney U rank sum test. (C) Calcium responses in OLQ to harsh head touch. Red traces indicate the average percentage change in R/R_0_ for selected genotypes from (B). Gray shading indicates SEM. None of these genotypes visibly altered the morphology of OLQ, or the expression pattern of the cameleon transgene. (D) Calcium responses in OLQ to nose touch. Red traces indicate the average percentage change in R/R_0_ for selected genotypes from (B). Gray shading indicates SEM.

If signaling in the nose touch circuit is mediated primarily by gap junctions, information flow through RIH might be bidirectional: just as activation of neurons such as OLQ can indirectly excite FLP, FLP activation could be able to excite OLQ. We examined this possibility by imaging OLQ calcium dynamics in response to mechanical stimuli sensed by FLP. We observed that harsh touch applied to the side of the head led to robust calcium transients in OLQ as well as RIH (Figures 8B and 8C; Figure S7E). Mutations in the mechanosensory channel *mec-10* eliminated OLQ and RIH responses to harsh head touch, and these responses could be rescued by FLP-specific expression of *mec-10* (Figures 8B and 8C; Figure S7E). Moreover, ablation of RIH eliminated the harsh head-touch-evoked calcium transients in OLQ (Figures 8B and 8C), indicating that the FLPs indirectly activate the OLQs through the RIH-centered network.

We also tested the effect of the network on nose touch responses in OLQ. Interestingly, a *mec-10* mutation significantly impaired OLQ and RIH calcium responses to nose touch; these defects were rescued by *mec-10(+)* expression in FLP (Figures 8B and 8D). Furthermore, ablation of RIH significantly reduced the responses of the OLQ neurons to nose touch (Figures 8B and 8D). These results indicate that just as the nose touch responses of the FLPs depend on a combination of RIH-mediated network activity and cell-autonomous MEC-10 function, OLQ nose touch responses depend on both RIH-mediated network activity and cell-autonomous OSM-9 function.

## Discussion

We have shown here how a network of interacting mechanosensory neurons detects nose touch stimuli and in response evokes escape behavior. Two classes of primary nose touch mechanoreceptors, the labial OLQ and cephalic CEP neurons, are required to indirectly facilitate gentle nose touch responses in the FLP head nociceptors. Nose touch activation of OLQ/CEP appears to excite the RIH interneuron through electrical synapses; this in turn depolarizes the FLP nociceptors, allowing these intrinsically high-threshold mechanoreceptors to respond to low-threshold nose touch stimuli. The FLPs most likely then activate the backward-command interneurons through synaptic connections to evoke reversal behavior. In a parallel pathway, the ASH polymodal nociceptors are likely to also excite the command interneurons in response to nose touch stimulation.

This model represents a significant revision in our understanding of the neural basis of nose touch perception in *C*. *elegans*. Previous cell-killing experiments identified ASH and FLP as the neurons whose ablation led to the most significant nose touch avoidance defects (Kaplan and Horvitz, 1993); on this basis, these two neuron pairs were thought to autonomously sense most nose touch stimuli (Driscoll and Kaplan, 1997). Because OLQ and CEP ablations had little or no effect on nose touch avoidance, these neurons were thought to be only weakly sensitive to nose touch and relatively unimportant for escape behavior. Our new data indicate that these neurons respond robustly to nose touch, and in doing so contribute to the nose touch response of FLP. Mutations affecting OLQ or CEP mechanosensory molecules significantly compromise nose touch avoidance and reduce nose-touch-evoked calcium transients in FLP. Through their RIH-mediated electrical coupling to FLP, active OLQ and CEP neurons appear to facilitate FLP activity, whereas inactive OLQ and CEP neurons appear to inhibit FLP. Collectively, the RIH-centered nose touch network may act as a kind of coincidence detector, by which coordinated activity of all the inputs facilitates responses throughout the circuit while lack of coordinated activity suppresses responses. These results highlight the importance of combining the use of in vivo recordings in combination with ablation experiments in dissecting neural circuit mechanisms.

The nose touch circuit we have defined here is similar in many ways to the recently described hub-and-spoke network controlling aggregation behavior in *C*. *elegans* (Macosko et al., 2009). In both cases, sensory information flows inward from the sensory neurons at the spokes to the integrating neuron at the hub. Processed information also flows outward through the gap junctional connections, with the spoke neurons playing a second role as behavior-specific outputs of the network. For example, the FLP neurons function both as polymodal nociceptor inputs to the circuit, as well as serving as the primary output from the RIH hub neuron to the command interneurons that execute the reversal reflex. The OLQ and CEP neurons appear to play similar dual roles as gentle touch mechanosensors and outputs for control of foraging and slowing behaviors. In this way, the network acts to couple distinct motor programs and allow their modulation by common sensory inputs.

The bidirectional nature of information flow in the network allows interconnected sensory neurons to modify and fine-tune each other's receptive properties. For example, over most of its receptive field, the FLP neurons respond only to high-threshold mechanical stimuli through its cell-autonomous MEC-10 harsh touch receptors. However, the electrical connectivity between FLP, OLQ, and CEP nose touch mechanoreceptors allows the threshold for touch sensitivity in FLP to be reduced when the CEP and OLQ neurons are active, facilitating responses to gentle nose touch. Thus, extrinsic network activity defines a gentle touch-sensitive region within the larger receptive field of FLP, which otherwise responds only to harsh touch. In this way, coordinated activity within the nose touch network is able to partially transform the FLPs from harsh touch to gentle touch sensors. Similarly, OLQ responses to nose touch are dependent on both the cell-autonomous activity of the OSM-9 TRPV channel as well as network inputs through RIH. Thus, lateral coupling between head mechanoreceptors allows sensory integration to occur at the most peripheral layer of the nose touch circuit, that of the sensory neurons themselves.

Hub-and-spoke electrical networks present certain problems for information processing by the nervous system. In particular, how can stimuli such as nose touch and harsh touch, which appear to activate most if not all neurons in the circuit, be distinguished? Differences in neuronal dynamics may play an important role; harsh head touch for example appears to evoke longer-lasting responses in OLQ and FLP than nose touch. The magnitudes of responses in different neurons also vary; harsh head touch responses are larger than nose touch responses in FLP but of similar size in OLQ. It will be interesting to explore how these factors influence the behavioral responses to these different stimuli.

The responses of sensory neurons are often considered to reflect the intrinsic properties of a cell and its sensory transduction pathways. However, the importance of interactions between sensory neurons in modifying these properties is becoming increasingly clear. In mammals, chemosensory neurons in taste buds are connected by both electrical and chemical synapses as well as by paracrine signaling (Huang et al., 2009; Dando and Roper, 2009). Likewise, extensive gap junction coupling has been shown to occur between many cell types in the retina, including rod and cone photoreceptors (Nelson, 1977). In at least some cases, the functions of these connections parallel those in the *C*. *elegans* nose touch circuit. For example, gap junctions between low-threshold rods and higher-threshold cones can facilitate responses in cone cells in low ambient light (Schneeweis and Schnapf, 1995), just as electrical connectivity in the nose touch circuit can facilitate gentle touch responses in the FLP nociceptors. Our finding that electrically mediated lateral interactions can tune the properties of sensory neurons in the nose touch circuits of *C*. *elegans* may suggest the existence of similar mechanisms in the nociceptive and somatosensory pathways of larger nervous systems.

## Experimental Procedures

A complete strain list and descriptions of plasmid and strain constructions are in Supplemental Experimental Procedures.

### Cell Ablations

Laser ablations were carried out using a standard protocol (Bargmann and Avery, 1995). The RIHs, OLQs, and FLPs were ablated in the early L1 stage, usually within 3–4 hr after hatching; the PVD cells were ablated at a slightly later stage, near the end of L1. Loss of the ablated cell was confirmed by observing loss of cameleon fluorescence in the adult animal.

### Calcium Imaging

Optical recordings were performed essentially as described (Kerr et al., 2000; Kerr, 2006) on a Zeiss Axioskop 2 upright compound microscope equipped with a Dual View beam splitter and a UNIBLITZ Shutter. Fluorescence images were acquired using MetaVue 6.2. Filter-dichroic pairs were excitation, 400–440; excitation dichroic 455; CFP emission, 465–495; emission dichroic 505; YFP emission, 520–550. Individual adult worms (∼24 hr past L4) were glued with Nexaband S/C cyanoacrylate glue to pads composed of 2% agarose in extracellular saline (145 mM NaCl, 5 mM KCl, 1 mM CaCl_2_, 5 mM MgCl_2_, 20 mM D-glucose, 10 mM HEPES buffer [pH 7.2]). Serotonin was also included at a concentration of 5 mM for nose touch-imaging experiments. Worms used for calcium imaging had similar levels of cameleon expression in sensory neurons as inferred from initial fluorescence intensity. Acquisitions were taken at 28 Hz (35 ms exposure time) with 4 × 4 or 2 × 2 binning, using a 63× Zeiss Achroplan water-immersion objective. Thermal stimulation was applied as described (Chatzigeorgiou et al., 2010b).

### Nose Touch Stimulation

The nose touch stimulator was a needle with a 50 μm diameter made of a drawn glass capillary with the tip rounded to ∼10 μm on a flame. We positioned the stimulator using a motorized stage (Polytec/PI M-111.1DG microtranslation stage with C-862 Mercury II controller). The needle was placed perpendicular to the worm's body at a distance of 150 μm from the side of the nose. In the “on” phase, the glass tip was moved toward the worm so that it could probe ∼8 μm into the side of the worm's nose on the cilia and held on the cilia for 1 s, and in the “off “ phase the needle was returned to its original position.

### Harsh Head Touch Stimulation

To visualize the harsh head touch response in FLP, the same nose touch setup was used, but the probe was aligned in a more posterior position between the two bulbs of the pharynx. The probe was displaced ∼24 μm at a raised speed of 2.8 mm/s. The stimulus was a buzz (i.e., the probe was displaced 2.5 μm in and out for the duration of the stimulus) lasting ∼1 s.

### Microscopy for Still Images

To obtain single images we used a Zeis LSM 510 Meta confocal microscope with a 40× objective. Images were exported as single TIFF files. To measure the intensity of the fluorescence, we imported the TIFF image in ImageJ. We measured the mean intensity of a region of interest encompassing the neuronal cell body using an arbitrary scale between 0 and 255.

### Nose Touch Behavioral Assays

For nose touch, assay plates were prepared fresh within 4 hr of use by spreading one drop of a saturated *E*. *coli* strain OP50 culture onto nematode growth medium plates. Two plates of ten worms each per genotype were allowed to move forward into an eyelash in the path of the worm. We recorded either a reversal response or null response. We scored the assay blinded and repeated it on at least 5 independent days. The nose-touch insensitive mutant *glr-1(n2461)* was used as a control.

## Supplemental Information

Document S1. Eight Figures and Supplemental Experimental Procedures
