# Supplementary material for: Lateral Facilitation between Primary Mechanosensory Neurons Controls Nose Touch Perception in C. elegans
Source: Neuron. 2011 Apr 28;70(2):299–309. doi: 10.1016/j.neuron.2011.02.046 (PMC3145979; doi:10.1016/j.neuron.2011.02.046)
Supplement: Document S1. Eight Figures and Supplemental Experimental Procedures [file mmc1.pdf]

Neuron, Volume *70*

Supplemental Information

Lateral Facilitation between Primary Mechanosensory Neurons Controls Nose Touch Perception in *C. elegans*

Marios Chatzigeorgiou and William R. Schafer

Inventory:

1. Supplemental Experimental Procedures
2. Legends to Supplemental Figures
3. Figure S1 (supporting data for Figure 1)
4. Figure S2 (supporting data for Figure 1)
5. Figure S3 (supporting data for Figure 2)
6. Figure S4 (supporting data for Figure 3)
7. Figure S5 (supporting data for Figure 3)
8. Figure S6 (supporting data for Figure 5)
9. Figure S7 (supporting data for Figure 6)
10. Figure S8 (supporting data for Figure 6)

SUPPLEMENTAL EXPERIMENTAL PROCEDURES

C. elegans Strains

Strains used:
AQ2145 *ljEx19[egl-46::YC2.3; lin-15(+)]*
AQ2045 *ljIs104[cat-1::YCD3]*
AQ906 *bzIs17[mec-4::YC2.12]*
AQ1490 *ljEx130[ocr-4::YCD3]*

AQ2695 *unc-13(e51); ljIs104[cat-1::YCD3]*

AQ2126 *mec-10(tm1552); ljEx19*
AQ2270 *mec-10(tm1552); ljEx19; ljEx220[egl-46::mec-10(genomic); unc-122::GFP]*
AQ2692 *mec-10(tm1552); ljEx381[egl-46::mec-10(cDNA); elt-2::RFP]*
AQ2693 *mec-10(tm1552); ljEx382[egl-46::mec-10(cDNA); elt-2::RFP]*
AQ2694 *mec-10(tm1552); ljEx381; ljEx19[egl-46::YC2.3; lin-15(+)]*
AQ2697 *mec-10(tm1552); ljIs104[cat-1::YCD3]*
AQ2698 *mec-10(tm1552); ljEx381; ljIs104[cat-1::YCD3]*
AQ2699 *mec-10(tm1552); ljEx130[ocr-4::YCD3]*
AQ2700 *mec-10(tm1552); ljEx381; ljEx130 [ocr-4::YCD3]*
AQ2481 *mec-10(tm1552);bzIs17; ljEx275[mec-4::mec-10(cDNA)]*
AQ1413 *mec-10(tm1552);bzIs17*
AQ2701 *mec-10(tm1552);bzIs17; ljEx381*

AQ2125 *trpa-1(ok999); ljEx19*
AQ1491 *tpra-1(ok999); ljEx130[ocr-4::YCD3]*
AQ2679 *trpa-1(ok999); ljEx373[ocr-4::trpa-1(cDNA); unc-122::GFP]*
AQ2680 *trpa-1(ok999); ljEx374[ocr-4::trpa-1(cDNA); unc-122::GFP]*
AQ2696 *trpa-1(ok999); ljEx374; ljEx130[ocr-4::YCD3]*
AQ2681 *trpa-1(ok999); ljEx374; ljEx19[egl-46::YC2.3]*
AQ2682 *trpa-1(ok999); ljEx374; ljIs104[cat-1::YCD3]*
AQ2702 *trpa-1(ok999); ljIs104[cat-1::YCD3]*
AQ1492 *trpa-1(ok999); ljEx115[del-2::trpa-1(cDNA); unc-122::GFP]; ljEx130*
AQ2265 *trpa-1(ok999); ljEx115; ljEx19[egl-46:;YC2.3]*
AQ2262 *trpa-1(ok999); ljEx19; ljEx119[sra-6::trpa-1(cDNA); unc-122::GFP]*

AQ2574 *unc-7(e5); ljIs104[cat-1::YCD3]*
AQ2683 *unc-7(e5); ljEx375[egl-46::unc-7(cDNA); cat-1::unc-7(cDNA); elt-2::RFP]*
AQ2684 *unc-7(e5); ljEx376[egl-46::unc-7(cDNA); cat-1::unc-7(cDNA); elt-2::RFP]*
AQ2685 *unc-7(e5); ljEx384; ljIs104[cat-1::YCD3]*
AQ2703 *unc-7(e5); ljEx19[egl-46::YC2.3]*
AQ2707 *unc-7(e5); ljEx384; ljEx19[egl-46::YC2.3]*

AQ2148 *osm-9(ky10); ljEx19[egl-46::YC2.3]*
AQ2149 *osm-9(ky10); mec-10(tm1552); ljEx19[egl-46::YC2.3]*
AQ2274 *osm-9(ky10); ljEx19; ljEx222[sra-6::osm-9(genomic); unc-122::GFP]*
AQ2294 *osm-9(ky10); ljEx19; ljEx235[del-2::osm-9(genomic); elt-2::RFP]*
AQ2686 *osm-9(ky10); ljEx377[egl-46::osm-9(cDNA); elt-2::RFP]*
AQ2687 *osm-9(ky10); ljEx378[egl-46::osm-9(cDNA); elt-2::RFP]*
AQ2688 *osm-9(ky10); ljEx377; ljEx19[egl-46::YC2.3]*
AQ2689 *osm-9(ky10); ljEx379[ocr-4::osm-9(cDNA); elt-2::RFP]*
AQ2690 *osm-9(ky10); ljEx380[ocr-4::osm-9(cDNA); elt-2::RFP]*
AQ2691 *osm-9(ky10); ljEx380; ljEx130[ocr-4::YCD3]*

AQ2284 *trp-4(ok1605); ljEx19[egl-46::YC2.3]*
AQ2266 *trp-4(ok1605); ljEx19; ljEx128[dat-1::trp-4(cDNA); unc-122::GFP]*
AQ2704 *trp-4(ok1605); osm-9(ky10); ljEx19[egl-46::YC2.3]*
AQ2705 *trp-4(ok1605); ljEx128; ljIs104[cat-1::YCD3]*
AQ2706 *ljEx383; Ex[dat-1::GFP]*

Generation of FLP/PVD cameleon line *ljEX19*

The *egl-46* promoter region was obtained from plasmid TU#307 (Wu et al., 2001), a gift from the lab of Martin Chalfie. A 3 kb HinDIII/NotI fragment was fused to cameleon YC2.3 in the vector pPD95.75 (A. Fire). Transgenic lines were obtained by germline injection of a *lin-15(n765)* mutant strain with the *egl-46::YC2.3* plasmid at a concentration of 50 ng/µl along with *lin-15(+)* genomic DNA (30 ng/µl) as a coinjection marker. Once a stable transgenic line was obtained, the *lin-15(n765)* allele was then removed by backcrossing to wild-type (N2) animals.

Transgenic rescue lines:

The *egl-46* promoter was obtained from plasmid TU#307 (Wu et al., 2001), a kind gift from the Chalfie Lab. Using the primers GGCCTTCTGAAATCAAAACG and AGTTCACGCCAGATGCAAGATG we PCR amplified a 3 kb fragment which was cloned into a Gateway 3-way P4-P1R donor vector. The *ocr-4* promoter we used was a 4.8kb fragment previsously described by Kindt et al (Kindt et al., 2007b). A 3 kb *sra-6*

promoter fragment cloned into a Gateway P4-P1R vector was obtained in the form of plasmid MGW1.2, a gift by E. Busch from the de Bono group. The 5' end of the promoter is defined by the sequnce 5'-CTTTTAGATATAATAAATCGAAATTG-3' while the 3' end is defined by the sequence 5'-GGCAAAATCTGAAATAATAAATATTAAATTC-3' adjacent of the start site of *sra-6*. A 2.4 kb *del-2* promoter was amplified using the following primers 5'-GATTGACATCTAGTAATTTTTAAG-3' and

5'-GCTGCAATTGAGTAGAACATGTAG-3'.

A *dat-1* promoter of length 0.8 kb was amplified. The 5' sequence was the following

5'-CTCTGAAATGTTTCTAGTCGTTTTTG-3' and

5'- GGCTAAAAATTGTTGAGATTCG-3' adjacent to the start site. A 4.4 kb fragment of *mec-10* genomic DNA was amplified uing Phusion High- Fidelity DNA Polymerase F-530S from adult genomic DNA using the primers 5'-GTACAAAATTCAAAAAATGAATCG-3' and 5'-GAAATAAGAAATTTATTTTCCG-3'. A 6.3 kb *osm-9* genomic DNA fragment was amplified from adult genomic DNA using the primers 5'-ATGGGCGGTGGAAGTTCGCGAAAC-3' and 5'-AAGAAAAAAAGTTTTCAAAAAATTAG-3'. We used a 5.6 kb *trp-4* cDNA clone (Kindt et al., 2007a), which was amplified from adult *C. elegans* RNA using the primers 5'-CCAGATGAATCCCCACACCTCCC-3' and

5'- AAGGGGTTATGCTAAAAACTAGTAGGTACTGC-3'. We obtained a 2.8 kb *osm-9* cDNA clone described in (Colbert et al., 1997), a gift from the Bargmann lab.

A *trpa-1* 3.6 kb cDNA fragment was amplified from a plasmid generated previously (Kindt et al., 2007b) using the primers 5'-ATGTCGAAGAAATCATTAGG-3' and 5'-TCAGTTATCTTTCTCCTCAAGT-3'. A 2.2 kb *mec-10* cDNA was amplified using Qiagen OneStep RT-PCR Kit from an RNA library using the primers 5'-ATGAATCGAAACCCGCGAATG-3' and 5'-TCAATACTCATTTGCAGCATTTTC-3'. In order to generate rescue plasmids, we used the MultiSite Gateway Three Fragment Vector Construction Kit (Invitrogen) to fuse promoter, ORF and an *unc-54* 3'UTR. All fragments generated by PCR were sequenced after cloning, and any mutations that were detected were rectified using the site-directed mutagenesis kit: QuickChange Lightning Site-Directed Mutagenesis Kit, from Stratagene.

We injected the *mec-10(tm1552); ljEx19[egl-46::YC2.3]* worms with *egl-46::mec-10(genomic)* at 80 ng/µl with *unc-122::GFP* at 25 ng/µl to generate array *ljEx220[egl-46::mec-10]*.

To generate arrays *ljEx381* and *ljEx382* we injected *mec-10(tm1552)* with *egl-46::mec-10(cDNA)* at 75 ng/µl and *elt-2::RFP* at 25 ng/µl. Two independent arrays were obtained and tested for rescue of the behavioral defect; one of these was subsequently crossed into the various calcium indicator strains that we used for imaging. To genotype the animals that came out of the crosses we used primers in the introns flanking the *tm1552* deletion. The "promoter only" controls for the *egl-46* promoter involved injection into a *mec-10(tm1552)* background in two different combinations. The first was *egl-46* promoter at 80 ng/µl with *unc-122::GFP* at 25 ng/µl and the other was *egl-46* promoter at 80 ng/µl with 25 ng/µl *elt-2::RFP*.

The *sra-6::osm-9* construct was injected in *osm-9(ky10); Ex19[egl-46::YC2.3]* at 50 ng/µl with *unc-122::GFP* at 20 ng/µl to generate array *ljEx222*. Similarly, the *del-2::osm-9(genomic)* clone was injected at 75 ng/µl together with *elt-2::RFP* at 25 ng/µl in *osm-9(ky10); Ex19[egl-46::YC2.3]* to obtain array *ljEx235*. To obtain cDNA rescue of *osm-9* in FLP, *osm-9(ky10)* animals were injected with *egl-46::osm-9(cDNA)* at 80ng/µl with 25 ng/µl of *elt-2::RFP*. Two independent arrays *ljEx377* and *ljEx378* were obtained and *ljEx377* was subsequently crossed into the various calcium indicator lines used for calcium imaging. To genotype the animals that came out of the crosses we used primers in the introns flanking the *ky10* point mutation to generate a PCR fragment which was subsequently sequenced with the primer CTAGGTGGAGGGCTGATTA. In order to obtain the rescue cDNA arrays *ljEx379* and *ljEx380* of *osm-9* in the OLQ neurons we injected *osm-9(ky10)* animals with 90 ng/µl *[ocr-4::osm-9(cDNA)]* and 25 ng/µl *elt-2::RFP*. To image from the rescue lines we crossed *ljEx380* with *osm-9(ky10) Ex[egl-46::YC2.3]*. The "promoter only" controls for the *egl-46* promoter involved injection into an *osm-9(ky10)* background at 80 ng/µl with *elt-2::RFP* at 25 ng/µl and for the *ocr-4* promoter at 90 ng/µl with 25 ng/µl *elt-2::RFP*. The "promoter only" controls were injected as follows: del-2 promoter was injected at 75 ng/µl with *elt-2::RFP* at 25 ng/µl , *egl-46* promoter involved injection of the promoter at 80ng/µl with 25 ng/µl of *elt-2::RFP* and the ocr-4 promoter at 90ng/µl with 25 ng/µl of *elt-2::RFP.* All empty promoters were injected in an *osm-9(ky10)* background.

The *ocr-4::trpa-1(cDNA)* construct was injected at 80 ng/µl together with *unc-122::GFP* at 25 ng/µl to create *ljEx373* and *ljEx374*. *ljEx374* was subsequently crossed into the various calcium indicator lines used for calcium imaging. *trpa-1(ok999); ljEx115[del-*

2::trpa-1(cDNA); unc-122::GFP] and *trpa-1(ok999); ljEx119[sra-6::trpa-1(cDNA); unc-122::GFP]* carrying animals (previously described in (Kindt et al., 2007b)) were crossed into the *ljEx19[egl-46::YC2.3]* to image from the FLP neurons. To genotype the animals that came out of the crosses we used primers in the introns flanking the *ok999* deletion. The "promoter only" controls were injected as follows: the *del-2* promoter was injected at 75 ng/µl with *unc-122::GFP* at 25 ng/µl and the *ocr-4* promoter at 80 ng/µl with 25 ng/µl of *unc-122::GFP.* All empty promoters were injected in a *trpa-1(ok999)* background.

To determine whether FLP and RIH nose touch defects in *trp-4(ok1605)* mutant worms were rescued by expression of *trp-4* in dopaminergic neurons we crossed *trp-4(ok1605); ljEx19* and *trp-4(ok1605); ljIs104* with *ljEx128[dat-1::trp-4(+); unc-122::GFP]* respectively*,* a previously described array carrying a *trp-4* cDNA expressed specifically in dopaminergic neurons (Kindt et al., 2007a). The "promoter only" control was injected as follows: *dat-1* promoter was injected at 20ng/µl with *unc-122::GFP* at 25 ng/µl in a *trp-4(ok1605)* background.

The *unc-7(e5)* allele was crossed with *ljIs104[cat-1::YCD3]* to image from RIH and with *ljEx19[egl-46::YC2.3].* To generate rescue lines for *unc-7* we co-injected *egl-46::unc-7(cDNA)* and *cat-1::unc-7(cDNA)* transgenes at a final concentration of 50 ng/µl each along with 25 ng/µl *elt-2::RFP*. Two independent arrays were obtained, *ljEx375* and *ljEx376*, and *ljEx375* was crossed into the appropriate imaging lines. In order to genotype the animals that came out of the crosses we used primers in the introns flanking the *e5*

point mutation to generate a PCR fragment which was subsequently sequenced. The "promoter only" controls were injected as follows: *cat-1* promoter was injected at 50 ng/µl with *elt-2::RFP* at 25 ng/µl, the *egl-46* promoter at 50ng/µl with 25 ng/µl of *elt-2::RFP and cat-1;elg-46* at a final concentration of *50* ng/µl with 25ng/µl *elt-2::RFP.* All empty promoters were injected in an *unc-7(e5)* background.

To generate the *dat-1::GFP* we injected the construct at 25 ng/µl with 25 ng/µl *unc-122::GFP*.

SUPPLEMENTAL REFERENCES

Colbert, H. A., Smith, T. L., and Bargmann, C. I. (1997). OSM-9, a novel protein with structural similarity to channels, is required for olfaction, mechanosensation, and olfactory adaptation in *C. elegans*. J Neurosci *17*, 8259-8269.

Hilliard, M. A., Bargmann, C. I., and Bazzicalupo, P. (2002). *C. elegans* responds to chemical repellents by integrating sensory inputs from the head and the tail. Curr Biol *12*, 730-734.

Kindt, K. S., Quast, K. B., Giles, A. C., De, S., Hendrey, D., Nicastro, I., Rankin, C. H., and Schafer, W. R. (2007a). Dopamine mediates context-dependent modulation of sensory plasticity in *C. elegans*. Neuron *55*, 662-676.

Kindt, K. S., Viswanath, V., Macpherson, L., Quast, K., Hu, H., Patapoutian, A., and Schafer, W. R. (2007b). *Caenorhabditis elegans* TRPA-1 functions in mechanosensation. Nat Neurosci *10*, 568-577.

Suzuki, H., Kerr, R., Bianchi, L., Frokjaer-Jensen, C., Slone, D., Xue, J., Gerstbrein, B., Driscoll, M., and Schafer, W. R. (2003). In vivo imaging of *C. elegans* mechanosensory neurons demonstrates a specific role for the MEC-4 channel in the process of gentle touch sensation. Neuron *39*, 1005-1017.

Wu, J., Duggan, A., and Chalfie, M. (2001). Inhibition of touch cell fate by *egl-44* and *egl-46* in *C. elegans*. Genes Dev *15*, 789-802.

SUPPLEMENTAL FIGURES

Figure S1: Supplemental data for touch-evoked calcium imaging experiments. A. Effect of cameleon transgenes on nose touch behavior. The cameleon imaging lines were assayed for their behavioural response to nose touch as described in methods. Fraction of animals responding is shown; error bars indicate SEM. 100 wild-type animals and 50 animals from each cameleon line were tested. **B. Protocols for nose touch and harsh touch stimulation.** Shown are images of animals receiving the nose touch or harsh head touch stimulus used in calcium imaging experiments. Worms were glued to the tip of the nose without covering the mouth on a 2% agarose pad and are subsequently immersed in extracellular saline. Two types of mechanical stimulation were delivered. A nose touch stimulus which consisted of a 1 second gentle (buzz) stimulus at the edge of the nose of the worm with a final displacement of 8 μm. A harsh head touch stimulus was delivered in a more posterior position near the terminal bulb of the pharynx. The probe was displaced a total of 24 μm into the worm's head.

A)

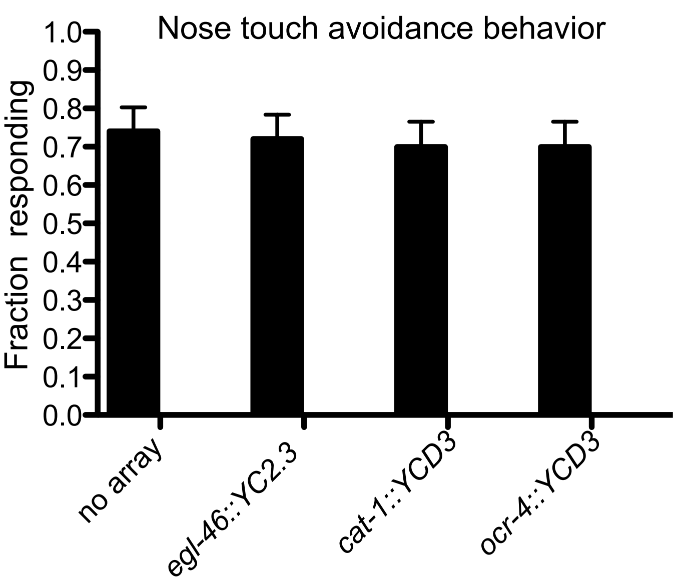

Nose touch avoidance behavior
Fraction responding
1.0
0.9
0.8
0.7
0.6
0.5
0.4
0.3
0.2
0.1
0.0
no array
egl-46::YC2.3
cat-1::YCD3
ocr-4::YCD3

B)

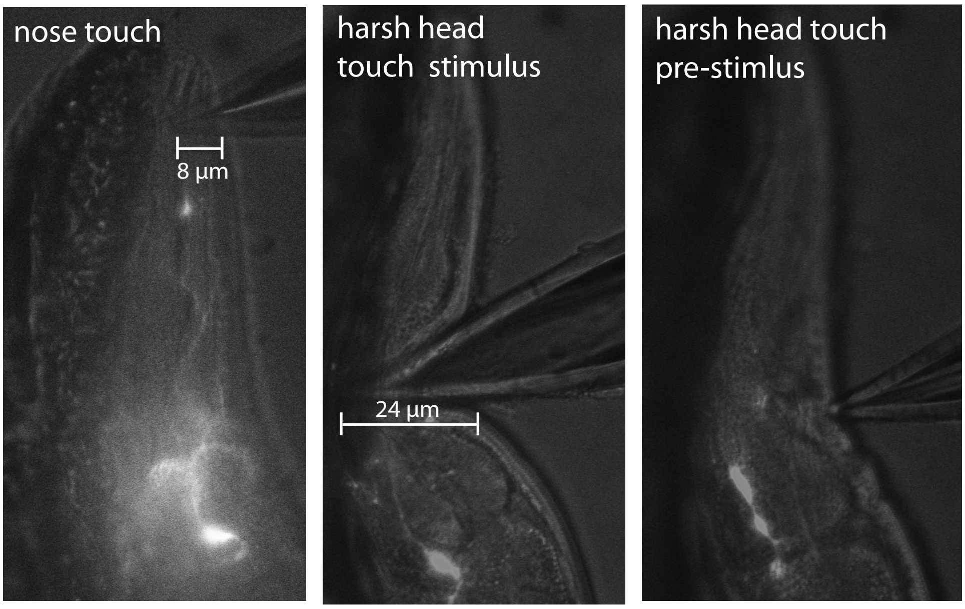

nose touch
8 μm
harsh head
touch stimulus
24 μm
harsh head touch
pre-stimlus

Figure S2: FLP heat responses in wild-type and mutant animals. A-C. Averaged calcium responses to heat shock stimulus. Each trace represents the average percentage change in R/R_0 for the indicated genotype, where R is the fluorescence emission ratio at a given time point and R_0 is its initial value. The green bar indicates a temperature of 20°C and the red bar indicates a period of stimulation at a temperature of 35°C. 18 wild-type, 10 *mec-10*, 10 *osm-9* and 10 *osm-9; egl-46::osm-9* animals were imaged for these experiments. **D. Scatter plot of peak calcium responses for each genotype.** Statistical significance (*** $p < .0005$) is according to the Mann-Whitney rank sum test.

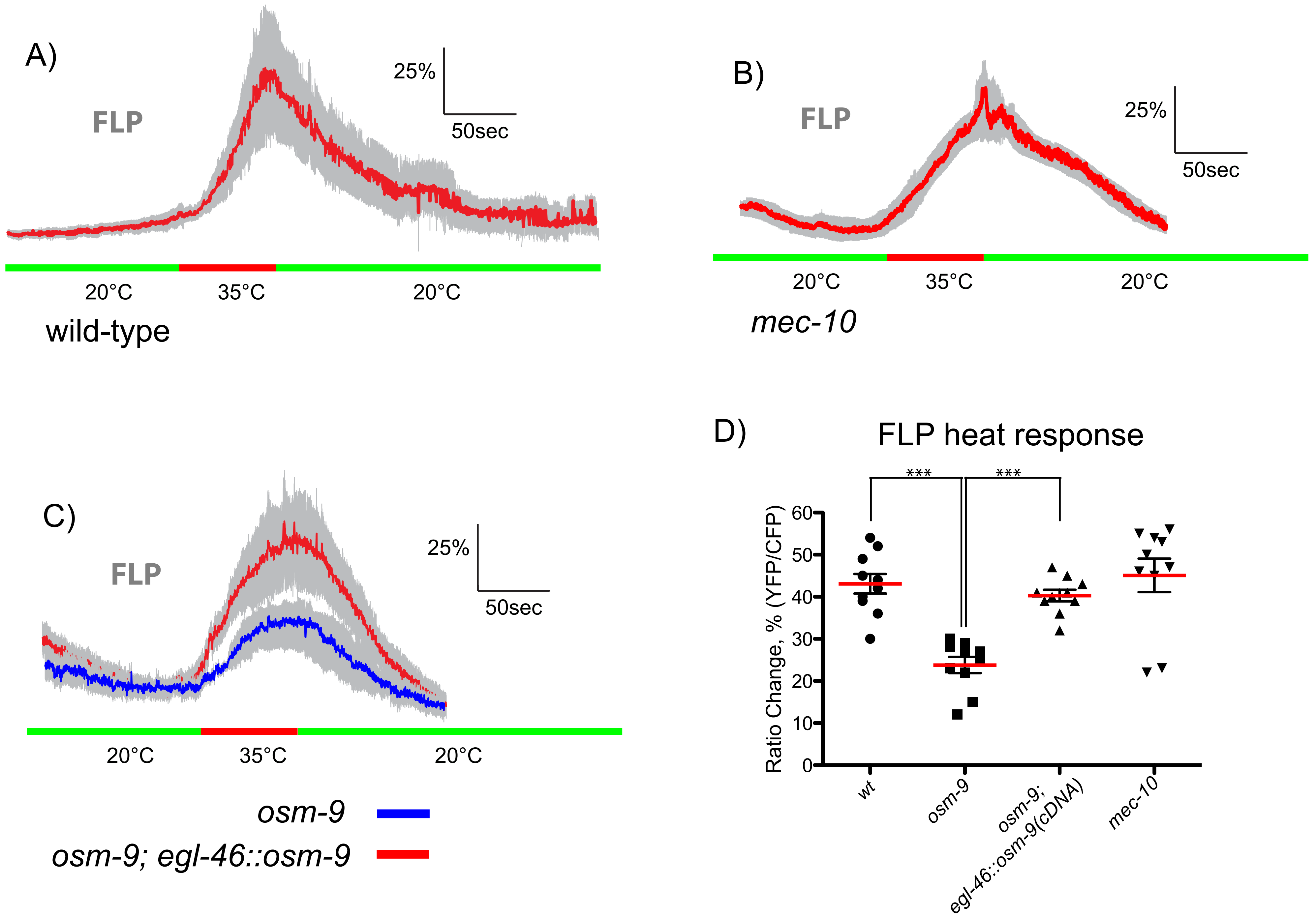

A)
FLP
25%
50sec
20°C
35°C
20°C
wild-type
B)
FLP
25%
50sec
20°C
35°C
20°C
mec-10
C)
FLP
25%
50sec
20°C
35°C
20°C
osm-9
osm-9; egl-46::osm-9
D)
FLP heat response
***
***
Ratio Change, % (YFP/CFP)
0
10
20
30
40
50
60
wt
osm-9
osm-9; egl-46::osm-9(cDNA)
mec-10

Figure S3: *egl-46::mec-10* does not rescue the *mec-10* touch defect in ALM neurons. A-D. Averaged calcium responses to gentle body touch in the ALMs. Animals expressing cameleon in the ALM gentle body touch neurons were given a 1 second buzz stimulus on the anterior body as described (Suzuki et al., 2003). Each red trace represents the average percentage change in R/R_0 for the indicated genotype, where R is the fluorescence emission ratio at a given time point and R_0 is its initial value. 11 animals per genotype were imaged. **E. Scatter plot of peak calcium respones for each stimulus**. The red line indicates mean ratio change; the error bars indicate SEM.

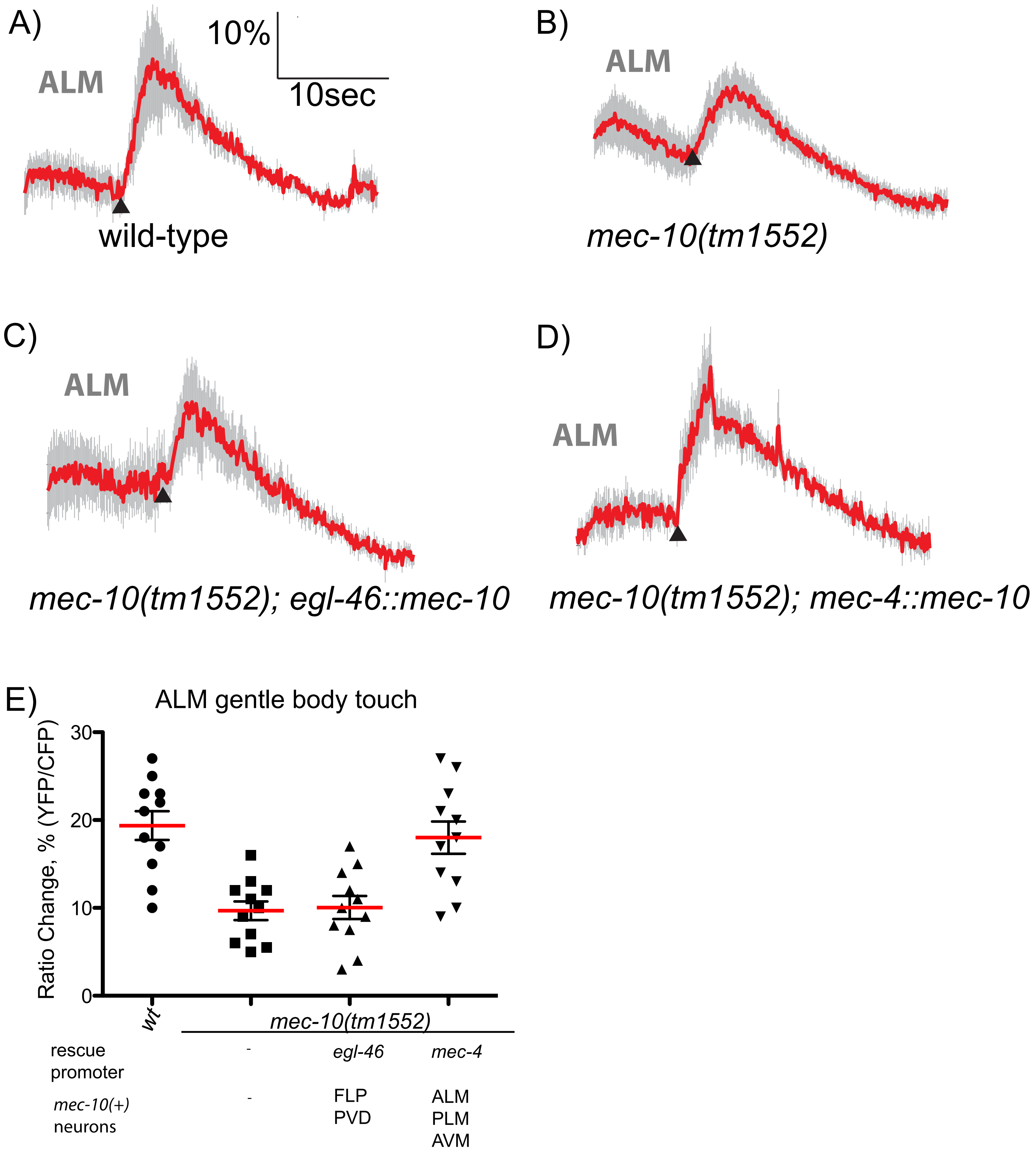

A)
10%
10sec
ALM
wild-type
B)
ALM
mec-10(tm1552)
C)
ALM
mec-10(tm1552); egl-46::mec-10
D)
ALM
mec-10(tm1552); mec-4::mec-10
E)
ALM gentle body touch
Ratio Change, % (YFP/CFP)
30
20
10
0
wt
mec-10(tm1552)
rescue promoter
-
egl-46
mec-4
mec-10(+) neurons
-
FLP
PVD
ALM
PLM
AVM

Figure S4: Effects of genetic background on expression levels of cameleon and promoter::GFP arrays. Mean fluorescence intensity was measured for the indicated neuronal cell body using a Zeiss LSM 510 Meta confocal microscope with a 40x objective. Images were exported as single TIFF files and fluorescence intensity was quantified using ImageJ. For cameleon lines the same strains used in imaging experiments in the main text were used. At least 5 animals of each genotype were analyzed.

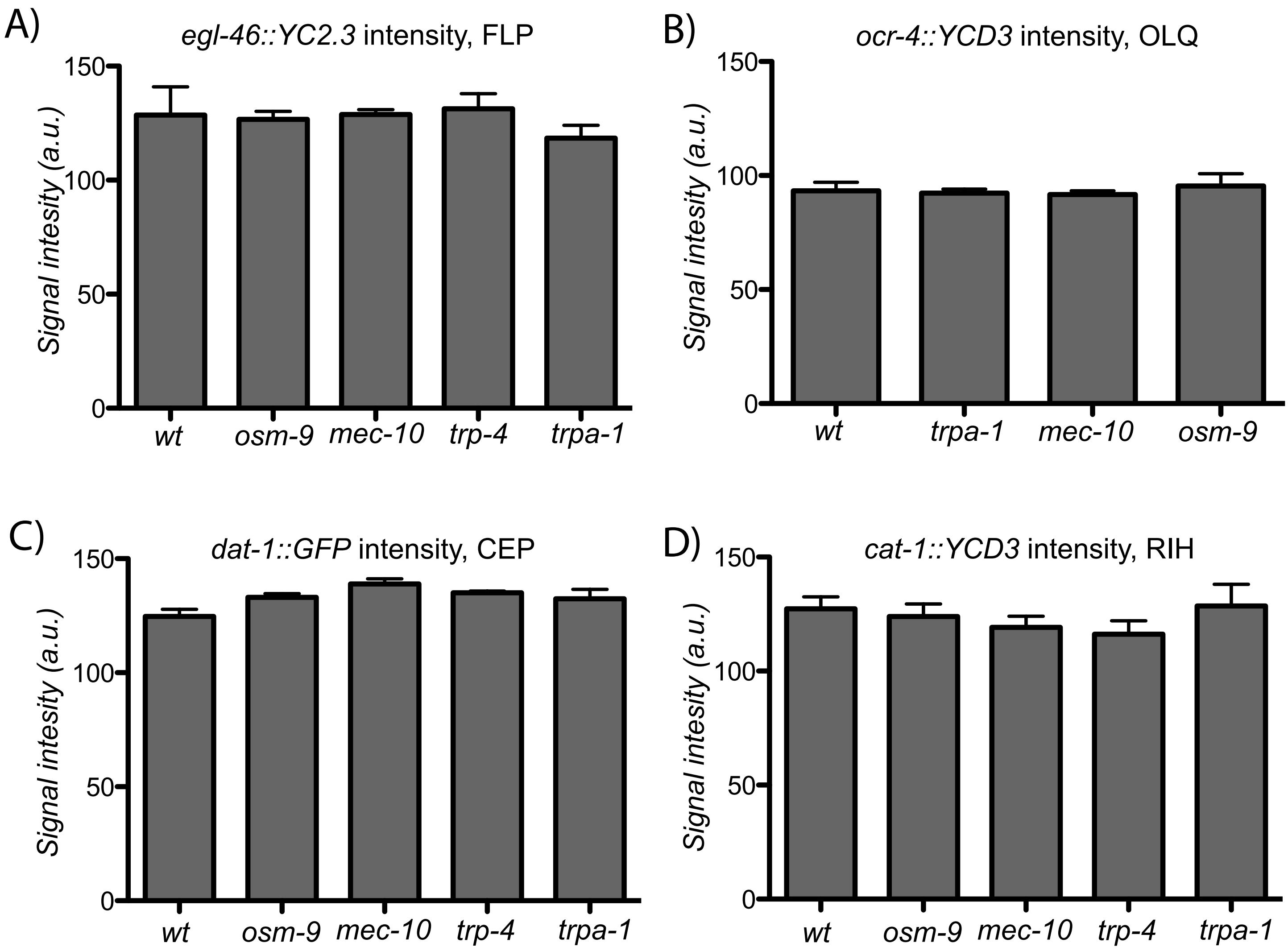

A)
egl-46::YC2.3 intensity, FLP
Signal intesity (a.u.)
150
100
50
0
wt
osm-9
mec-10
trp-4
trpa-1
B)
ocr-4::YCD3 intensity, OLQ
Signal intesity (a.u.)
150
100
50
0
wt
trpa-1
mec-10
osm-9
C)
dat-1::GFP intensity, CEP
Signal intesity (a.u.)
150
100
50
0
wt
osm-9
mec-10
trp-4
trpa-1
D)
cat-1::YCD3 intensity, RIH
Signal intesity (a.u.)
150
100
50
0
wt
osm-9
mec-10
trp-4
trpa-1

Figure S5: *sra-6::osm-9* rescues ASH-mediated osmotic avoidance behavior. Wild-type, *osm-9(ky10)*, and *osm-9(ky10); sra-6::osm-9(genomic)* animals were tested for escape behavior evoked by 1M glycerol as described (Hilliard et al., 2002). Each data point represents 50 worms assayed on three independent days. Error bars show S.E.M. *** indicates a significant difference between mutant and rescued strains according to the chi-square test ($p < 0.001$).

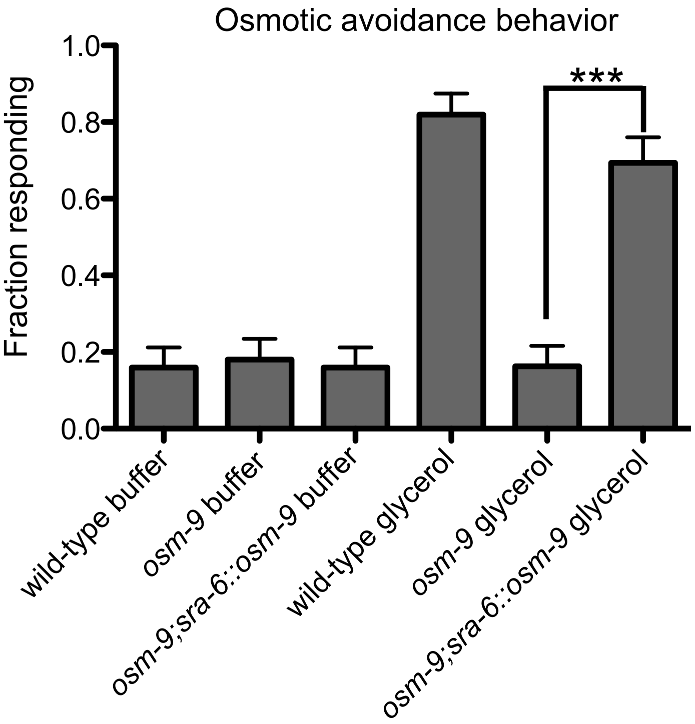

Osmotic avoidance behavior
Fraction responding
1.0
0.8
0.6
0.4
0.2
0.0
***
wild-type buffer
osm-9 buffer
osm-9;sra-6::osm-9 buffer
wild-type glycerol
osm-9 glycerol
osm-9;sra-6::osm-9 glycerol

Figure S6: *trpa-1* acts cell-autonomously in OLQ to promote nose touch response. Worms expressing cameleon in OLQ neurons were immobilized to the nose with glue and covered in extracellular saline containing 2 mM serotonin. A glass probe delivered a gentle buzzing stimulus to the nose. A-D Average traces in OLQ in response to 2nd nose touch stimulation (5 min after the first stimulation) in (A) wild-type, (B) *trpa-1*, (C) *trpa-1* with RIH ablated and (D) *trpa-1; ocr-4::trpa-1*. (E) Scatter plot quantifying the 2nd OLQ nose touch responses in wild-type (n=14), *trpa-1* (n=14), and *trpa-1; ocr-4::trpa-1* (n=13).

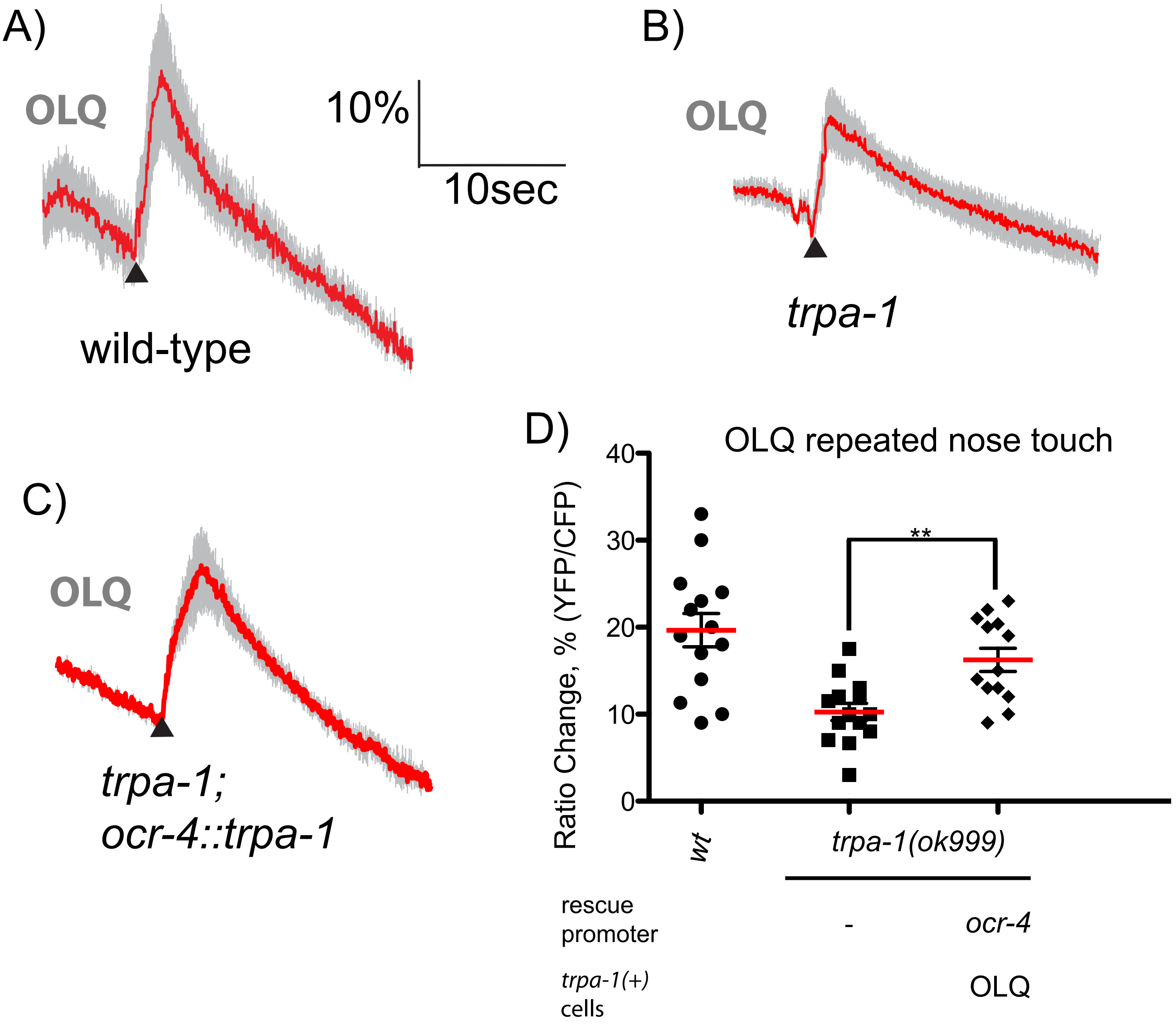

A)
OLQ
10%
10sec
wild-type
B)
OLQ
trpa-1
C)
OLQ
trpa-1;
ocr-4::trpa-1
D)
OLQ repeated nose touch
Ratio Change, % (YFP/CFP)
40
30
20
10
0
**
wt
trpa-1(ok999)
rescue
promoter
-
ocr-4
trpa-1(+)
cells
OLQ

Figure S7: Effect of innexin mutations on nose touch responses. A. Averaged responses of RIH neurons of *unc-7* innexin mutant and rescued animals animals to nose touch stimulation. Each solid trace represents the average percentage change in R/R_0 for 14 (wild-type, red trace) or 10 (*unc-7(e5)*, green trace) individual recordings. Gray shading indicates SEM of the mean response. Scale bars are indicated. None of these genotypes visibly altered the morphology of FLP or RIH or the expression patterns of the cameleon transgenes. **B. Averaged responses of FLP neurons of *unc-7* innexin mutant and rescued animals to nose touch stimulation.** Each solid trace represents the average percentage change in R/R_0 for 10 (wild-type, red trace) or 10 (*unc-7(e5)*, green trace) individual recordings. **C. Scatter plot of peak calcium responses.** In addition to the genotypes in panels A and B, 14 animals were imaged for RIH nose touch and 10 animals were imaged for FLP nose touch using *unc-7; egl-46::unc-7; cat-1::unc-7* rescue lines carrying the *cat-1::YCD3* and *egl-46::YC2.3* arrays. Statistical significance (*** $p < .0005$) is according to the Mann-Whitney rank sum test. **D. Effect of *unc-7* innexin mutations on nose touch-evoked escape responses.** 100 animals of each genotype were scored for reversals following nose touch stimulation. Statistical significance ($p < 0.005$) is according to the Student's t test. **E. Scatter plot of peak RIH calcium responses to harsh touch.** 10 animals were imaged for each genotype. Statistical significance (*** $p < .0005$) is according to the Mann-Whitney rank sum test.

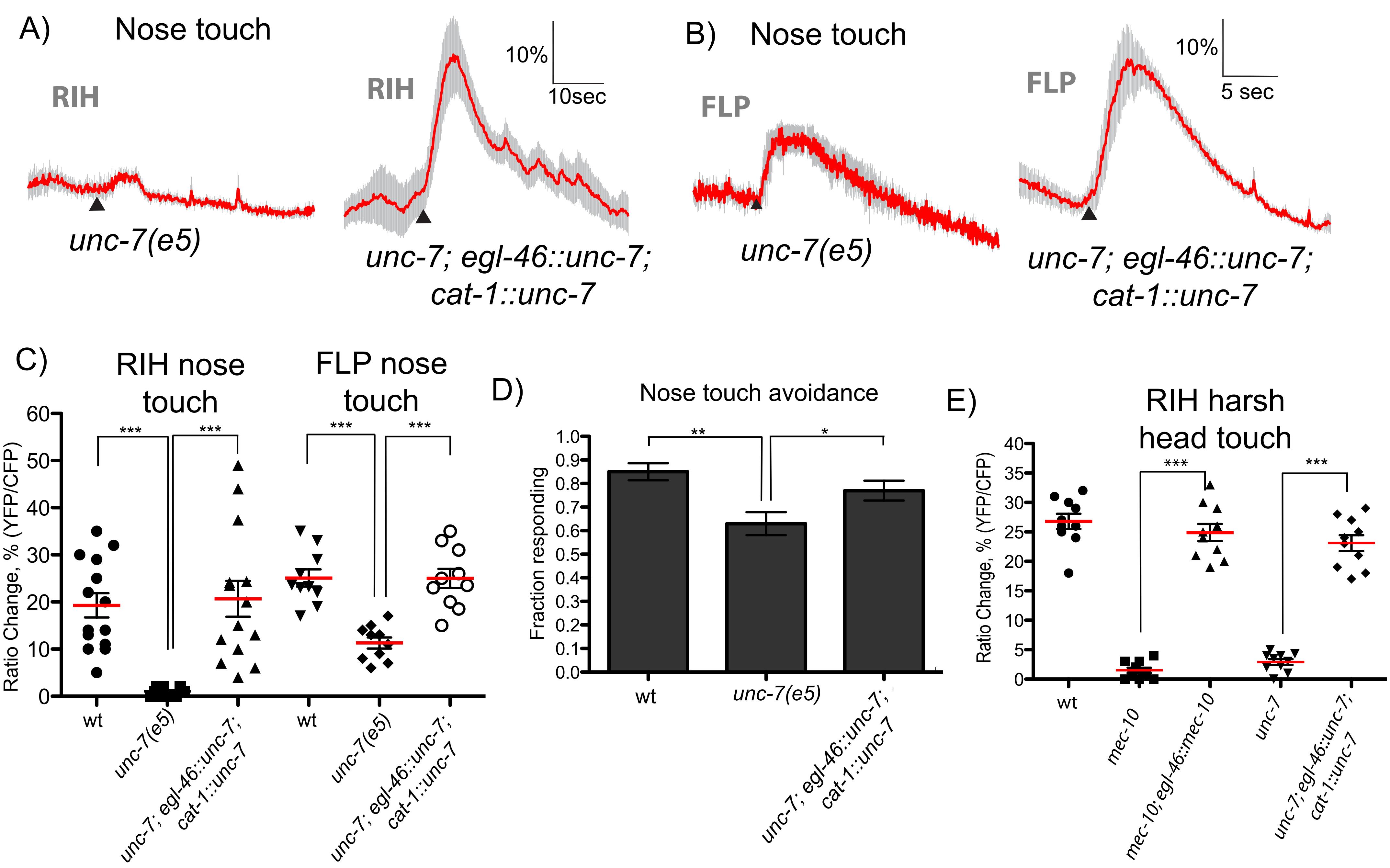

A)
Nose touch
RIH
unc-7(e5)
RIH
10%
10sec
unc-7; egl-46::unc-7; cat-1::unc-7
B)
Nose touch
FLP
unc-7(e5)
FLP
10%
5 sec
unc-7; egl-46::unc-7; cat-1::unc-7
C)
RIH nose touch
FLP nose touch
Ratio Change, % (YFP/CFP)
0
10
20
30
40
50
60
***
***
***
***
wt
unc-7(e5)
unc-7; egl-46::unc-7; cat-1::unc-7
wt
unc-7(e5)
unc-7; egl-46::unc-7; cat-1::unc-7
D)
Nose touch avoidance
Fraction responding
0.0
0.1
0.2
0.3
0.4
0.5
0.6
0.7
0.8
0.9
1.0
**
*
wt
unc-7(e5)
unc-7; egl-46::unc-7; cat-1::unc-7
E)
RIH harsh head touch
Ratio Change, % (YFP/CFP)
0
5
10
15
20
25
30
35
40
***
***
wt
mec-10
mec-10;egl-46::mec-10
unc-7
unc-7;egl-46::unc-7; cat-1::unc-7

Figure S8: Empty vector controls for *unc-7* nose touch rescue. A. Scatter plot of responses to nose touch in the RIH neuron. "Empty" plasmids containing only the indicated promoters were injected into *unc-7; cat-1::YCD3* animals at the same concentrations as the rescue constructs in Figure S7. 9 animals were imaged for each control strain. **B. Nose touch escape behavior** "Empty" plasmids containing only the indicated promoters were injected into *unc-7* animals at the same concentrations as the rescue constructs in Figure S7. 100 animals were assayed on 3 independent days. Error bars indicate SEM.

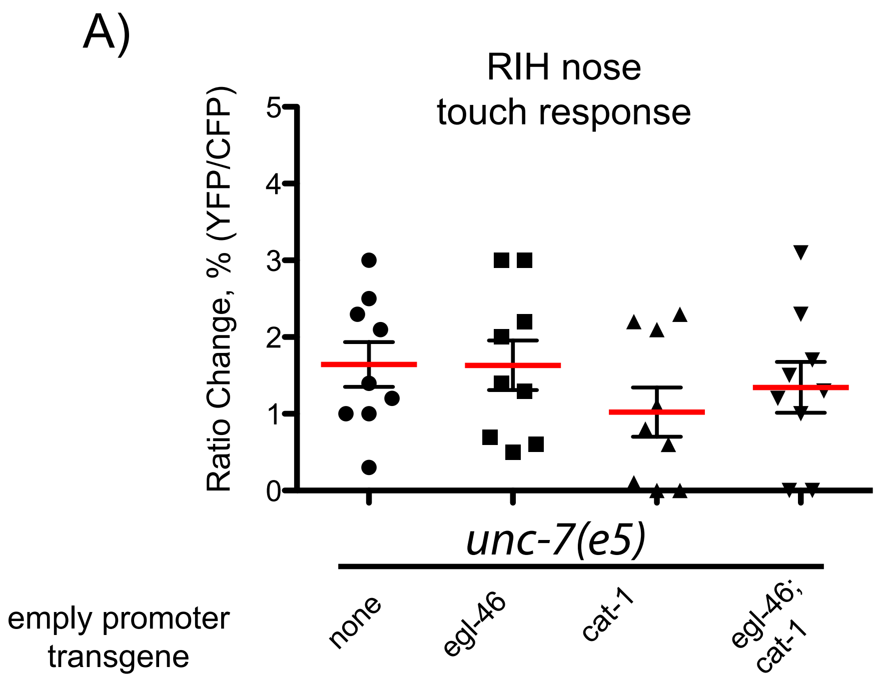

A)
RIH nose
touch response
Ratio Change, % (YFP/CFP)
0
1
2
3
4
5
unc-7(e5)
emply promoter
transgene
none
egl-46
cat-1
egl-46;
cat-1

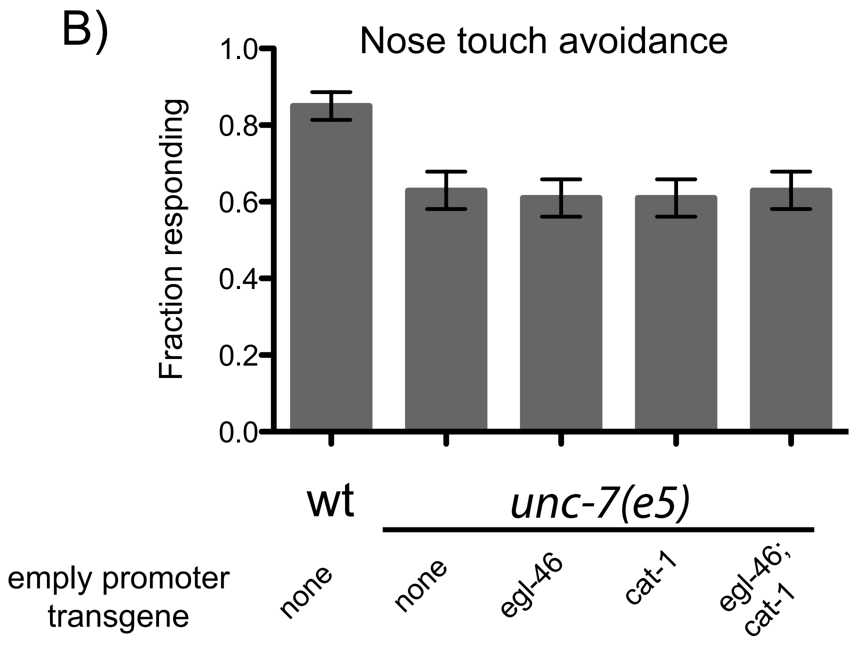

B)
Nose touch avoidance
Fraction responding
0.0
0.2
0.4
0.6
0.8
1.0
wt
unc-7(e5)
emply promoter
transgene
none
none
egl-46
cat-1
egl-46;
cat-1
